# Statistical optimization characterizations and Eco- friendly synthesis of silica from sugarcane bagasse

**DOI:** 10.1038/s41598-025-89366-6

**Published:** 2025-03-12

**Authors:** Girma Assefa Habte, Tafere Aga Bullo, Yasin Ahmed

**Affiliations:** School of Chemical Engineering, Jimma University, Jimma Institute of Technology, P.O. Box 378, Jimma, Ethiopia

**Keywords:** Central Composite Design, Sugarcane bagasse, Silica synthesis, Sol-gel, Chemical engineering, Materials science

## Abstract

This research explores the synthesis and optimization of Silica have been effectively produced from sugarcane bagasse (SB) using the sol-gel methods. Due to its rich silica content, sugarcane bagasse can be utilized as a viable alternative source for silica synthesis. Employing Central Composite Design, the study systematically varied combustion temperature (500–800 °C), combustion time (2–4 h), and digestion time (1–3 h) to enhance silica yield. The optimal conditions identified were a combustion temperature of 583.48 °C, a combustion time of 3.482 h, and a digestion time of 2.283 h, resulting in a silica yield of 69.6%. Comprehensive characterization of the synthesized silica was conducted through Fourier Transform Infrared Spectroscopy (FTIR), X-ray Diffraction (XRD), Scanning Electron Microscopy (SEM), Brunauer, Emmett, Teller model (BET) analysis and Thermo-gravimetric Analysis (TGA). XRD results indicated the amorphous nature of the silica, with a broad peak at 22.36°, akin to that of commercial silica. FTIR analysis revealed six characteristic peaks at wavenumbers corresponding to those found in commercial silica, confirming the presence of similar chemical groups. SEM imagery illustrated a disordered arrangement of silica with undefined morphology. The TGA analysis shows high thermal resistivity of silica with only 9% weigh loss at 800 °C. Overall, this study demonstrates that high-quality silica can be produced from sugarcane bagasse with minimal chemical input and energy consumption and highlighting its potential for diverse applications.

## Introduction

Silica is a versatile inorganic substance and a fundamental material in industries such as plastics, cosmetics, electronics, coatings, and rubber manufacturing^1^. Silica plays a role in environmental applications such as photo-catalysis^2^, adsorption of heavy metals^3,4^, color removal in water treatments^5^, and carbon dioxide-capturing applications^6^.

Commercially, silica can be produced from alkyl orthosilicates such as poly ethyl di-orthosilicate (PEOS), tetraethyl orthosilicate (TEOS), and tetramethyl orthosilicate (TMOS) ore using the appropriate catalysts^7^. These conventional precursors are expensive, difficult to obtain, or involve environmentally impactful processes^6^. Another typical silica precursor is sodium silicate, which is produced through an expensive process that requires heating quartz sand and sodium carbonate at high temperature of 1300 °C to 1500 °C^8^ and fusing it with sodium carbonate to form sodium silicate^9^. This leads to the emission of liquid effluents as well as a significant amount of carbon dioxide. Silica from sodium silicate is expensive due to high material costs^10^. Conventional precursors such as quartz sands and alkyl orthosilicates are energy-intensive, expensive, toxic, and non-eco-friendly productions that increase environmental stress^7^.

According to^11^, agricultural waste, such as rice husks, groundnut shells, bamboo leaves, wheat husks, rice straws, wheat straws, sugarcane leaf and bagasse, maize cobs, and others are considered promising sources of silica. The ash obtained from burning biomass waste contains inorganic residues mainly silica (SiO_2_) ranging from 50 to 90%^12^. These agricultural wastes are attractive due to their low raw material costs, high silica content, comparable silica grade, amount of energy content, and the presence of fine-sized amorphous material^12^.

In Ethiopia, sugarcane bagasse has less economic value except for its conventional uses as fuel for the boiler and for making boards. The disposal of this sugarcane bagasse in the sugar industry has covered large areas. It is also placed for long times in the open field. It leads to the growth of microorganisms like bacteria and attracts many insects, which may cause health problems for society^13^. As a result, converting bagasse into useful products like silica plays a significant role in sustainable raw material usage and environmental protection.

In this study the sustainable silica production from agricultural waste sources, specifically from sugarcane bagasse by establishing optimum production conditions. So, bio-silica from agricultural waste like sugarcane bagasse is used as a renewable and locally available source. Despite this, silica production parameters are optimizing the combustion temperature, combustion time, and digestion time in contrast to conventional approaches that mostly rely on empirical trial-and-error. It also helpful to develop appropriate models which correlate the synthesis parameters with the yield. The yield and silica extraction efficiency improved by this technique. This optimization process is essential for achieving the desired yields and properties in the produced silica. Various analytical techniques, including X-ray *Diffraction* (XRD), scanning electron microscope (SEM), and Fourier transform infrared (FTIR) spectroscopy have been utilized to characterize the produced silica and provide a comprehensive understanding of its morphology, surface structure, surface area, and functional groups of synthesized silica. The synthesized Silica was used for wastewater treatment.

## Methods

### Chemicals and materials used

The chemicals used in this thesis were citric acid to wash sugarcane bagasse, citric acid, and water used to wash the sugarcane bagasse and remove soluble impurities, and sodium hydroxide (98%) was employed to digest silica from sugarcane bagasse ash to form sodium silicate (sol). Hydrochloric acid (37% HCl) was utilized to precipitate silica from sodium silicate (sol) solution. All chemicals used were analytical grade.

### Raw material collections and proximate analysis

The Sugarcane bagasse was collected from the Wanji Shewa sugar factory using a plastic bag and chopped with a knife to reduce size. Then proximate analysis was carried out to identify the main contents of the raw material (sugarcane bagasse). It was performed by using ASTM standard procedure. The ASTM D-3173, ASTM-D3175, and ASTM D-3174 standard procedures were used to determine the moisture content, volatile matter content, and ash content of sugarcane bagasse respectively^14^. The fixed carbon content (FC) of the sugarcane bagasse was estimated using mass balance. FC was calculated by subtracting the total of the moisture content, volatile matter content, and ash content percentages from 100%.

### Bagasse pre-treatment

**Fig. 1:**
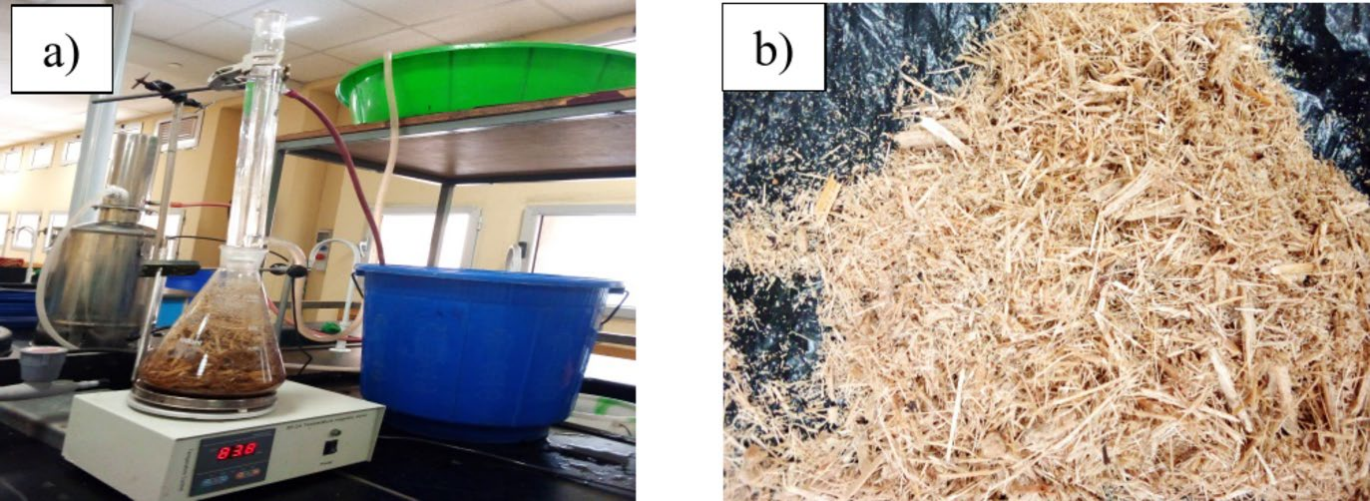
(**a**) Bagasse treatment by reflux condenser (**b**) acid-treated bagasse.

Figure 1 shows the collected bagasse was pretreated with water and organic acid. To eliminate soluble particles, dust, and other sand particles, sugarcane bagasse was extensively washed with water. It was subsequently rinsed with tap water and dried in a 105 °C oven for 12 h. The sugarcane bagasse was then treated with a citric acid solution with a 1:10 Solid/Liquid ratio to eliminate dissolved materials and metallic impurities and to partially hydrolyze the organic component before combustion. In a round bottom flask, 60 g of dry water-washed sugarcane bagasse was mixed with 600 ml of 1 M citric acid solution and cooked using a reflux condenser at 80 ℃ for 2 h. The solids were rinsed several times with distilled water to eliminate the acid retained after separation by settling and filtration and then dried at 105 °C for 12 h.

### Sugarcane bagasse ash preparation

**Fig. 2:**
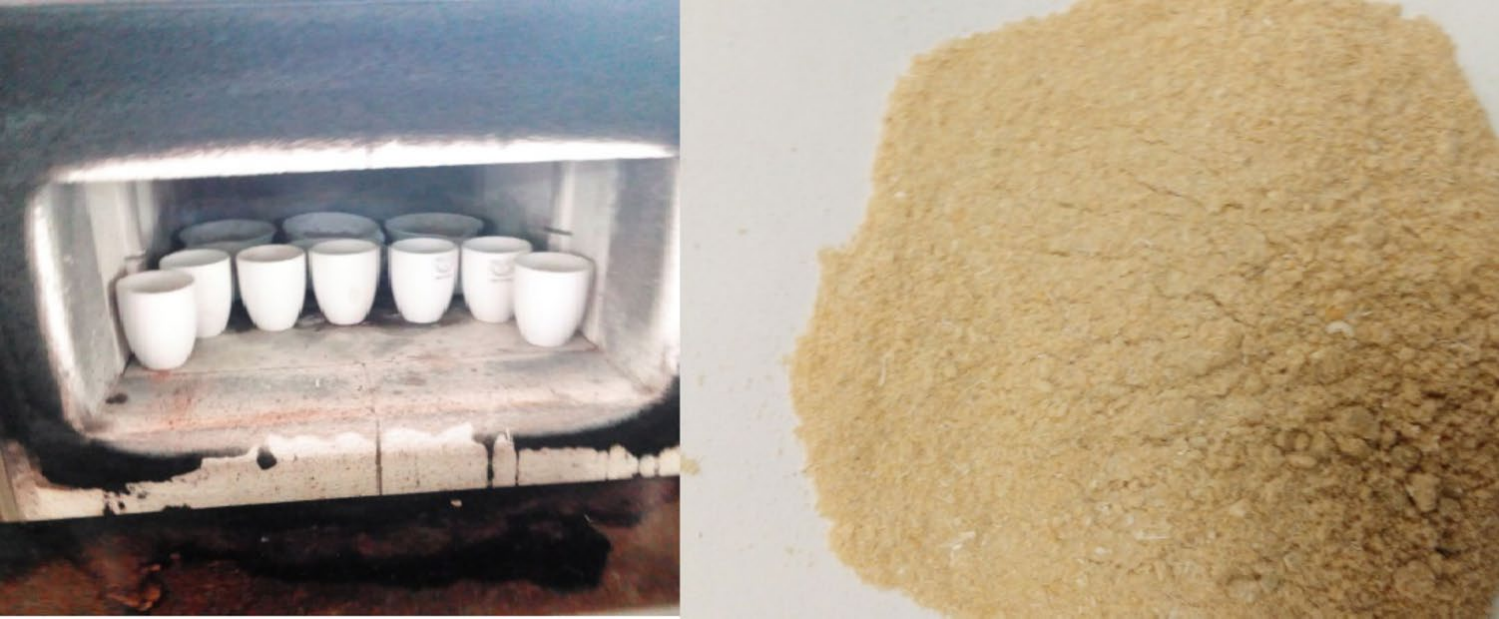
Ash preparation.

The combustion/incineration of pre-treated sugarcane bagasse (SCBs) was performed in the Material Science and Engineering laboratory using a muffle furnace with a temperature range of 500–800 ℃ for 2–4 h. The dried, acid-washed bagasse was placed in a crucible and subjected to a furnace at the predetermined temperature and duration outlined in the experimental design. Afterward, the crucibles were carefully removed using tongs and allowed to cool for one hour in desiccators. Figure 2 represents the resulting ashes preparation were labeled and stored for silica synthesis.

### Silica synthesis by sol-gel method

**Fig. 3:**
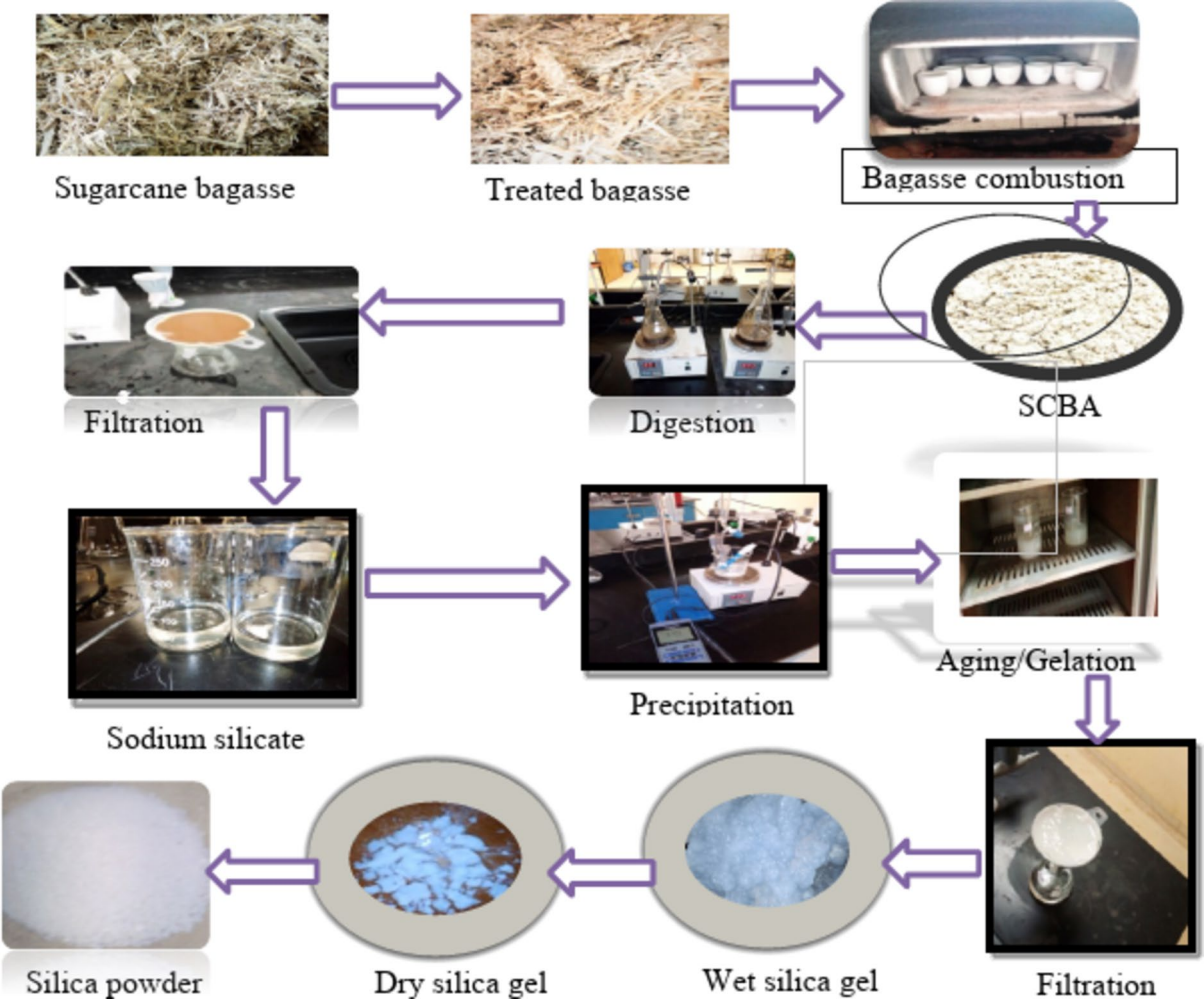
Flow diagram of laboratory work (experimental works).

Figure 3 represents the overall flow diagram of Silica synthesis from SCBA were digested (leached) with 2 M of NaOH solution to form sols. The digestion process was done by a 1:10 SCBA/NaOH (g/ml) ratio. In the beaker, 5 g of bagasse ash was combined with 50 ml of 2 M NaOH and placed on the hot plate for 2 h at 80 ℃. Constant stirring at 200 rpm was utilized to make a sodium silicate solution. The silica from the ash combines with sodium hydroxide to form a silicate (sol) solution. The reaction is given^15^ Eq. (1).

1 $$\:Ash+2NaOH\to\:{Na}_{2}{SiO}_{3}+{H}_{2}O$$

To eliminate solid residue the suspension was filtered via filter paper. The filtrate solutions contained silica supernatant, which was used as a silica precursor. The filtrate (sol) was progressively titrated with 1 M HCl, to precipitate the dissolved silicate into white gelatinous silica (SiO_2_) with steady stirring. The gel formation (precipitation) began at a pH of 10.5 and was continued by adding HCl dropwise until the solution’s pH reached 7. As stated^16^, further precipitation did not occur after the pH reached 7. According to^15^, the precipitation of sodium silicate was presented using the reaction given in Eq. (2).

2 $$\:{Na}_{2}{SiO}_{3}+HCl\to\:{SiO}_{2}+NaCl+{H}_{2}O$$

The precipitated solution that forms silica gel was taken into an incubator at 45℃ for 20 h of aging time. This process allowed for complete gel formation and to maintain the required silica gel properties. Subsequently, the gel underwent filtration through filter paper, with the solid residues (silica) being thoroughly rinsed with distilled water multiple times to eliminate any soluble particles and residual chemicals. Finally, the white solid (silica) was dried in an oven at 105 ℃ for 12 h before being labeled and then placed in a desiccator for further use.

### Silica yield determination

Silica yield was calculated to identify the optimum values of process parameters (combustion temperature, combustion duration, and digesting time) and to get the effect of each variable on the final yield. During the experiment, 5-gram ash was used for silica synthesis during each run. The silica yield from SCBA was determined using Eq. (3).

3 $$\:Yield\:\left(\%\right)=\frac{{M}_{{SO}_{2}}}{{M}_{SCBA}}$$

Where: M_SO2_ = mass of silica; M_SCBA_ = mass of SCBA.

### Experimental design and factors affecting the synthesis of silica

The Central Composite Design (CCD), which is a widely used application of Response Surface Methodology (RSM) or Design-Expert software 13.0 (Stat-Ease, Inc., Minneapolis), was used for the operational parameters of silica synthesis.

The experiments were designed by CCD, which can allow efficient estimation of terms in the regression model. It can be used to solve the internal as well as external effects of the variable based on the alpha value. The experiment aimed to determine the single effect and combined effect of combustion temperature, Combustion time, and digestion time on silica yield.

According to^11^, the average temperature range for agricultural waste burning to produce amorphous silica has been 500–800 ℃ and a digestion time of 2–4 h. The combustion temperature and time variations have affected the morphology and yield of the produced silica. It also affects the surface areas of the porous material. Digestion temperature has been used at 80 ℃ and with digestion time between 1 and 3 h which affects silica yield. PH of precipitation has been fixed at 7^17^. The experiment consisted of a total of 20 runs, determined by the standard formula for experimental design involving three factors, each at two levels, as indicated in Eq. (4).

4 $$\:Number\,of\,total\,run=2^k+2K+K_e$$

Where k is the number of independent factors and k_c_ number of central points (6). The factors selected and their levels to have been analyzed are shown in Table 1 below.

**Table 1:** Silica production parameters and their level of optimization.

No	Parameters	Lower level	Higher level
1	Combustion temperature	500	800
2	Combustion time	2	4
3	Digestion time	1	3

## Characterization of sugarcane bagasse ash and synthesis of silica

### X-ray diffraction

The XRD evaluation of SCBA and synthesis silica from SCBA was performed in the Jimma University, Jimma Institute of Technology, Faculty of Material Science and Engineering laboratory by using an XRD instrument (XRD-7000, Drawell, Shanghai, China) with a scanning range and rate of 10⁰-70⁰ and 0.0012⁰-70⁰ per minute respectively with Cu-kα (3KW) radiation X-ray source. To identify the crystallite nature of synthesized silica, the crystallite index (CI) has been calculated using the crystalline area and total area as indicated in Eq. (5).

5 $$\:CI=\left(\frac{Crystaline\:area}{Crystaline\:area+amorphous\:area}\right)100\%$$

### FTIR spectroscopy analysis

An infrared radiation (IR) test was used to determine surface functional groups that exist in the powder of SCBA and the synthesized silica. This was conducted in the laboratory of the Faculty of Material Science and Engineering at Jimma University Institute of Technology, by using (spectrum two models, PerkinElmer, Netherlands) which have a wavenumber range of 8300 –350 cm^− 1^. FTIR spectrum was used to identify the primary functional groups present in the raw material and product. The analysis was performed in the band spectra between wavenumber of 4000 m^− 1^ to 400 cm^− 1^.

### Scanning electron microscope (SEM) analysis

The SEM imaging analysis was carried out at the Department of Biology at Adama Science and Technology University (ASTU). The signals produced by SEM devices (GCM-6000 plus model) analysis create a two-dimensional image and provide details about the sample, such as the arrangement of particles and its exterior morphology (texture).

### Brunauer, Emmett, Teller model (BET) analysis

The BET analysis was performed at Bahirdar university chemical engineering laboratory. The specific surface area of the produced silica was determined by using Nova station B equipment by taking 0.05 g of sample. The BET surface area was measured by N_2_ adsorption-desorption at 77 K using the gas adsorption system. Pore volume was calculated from the base of adsorbed N_2_.

### Thermo-gravimetric (TGA) analysis

The thermal stability of the silica sample was assessed using a DTG-60 H differential thermal gravimetric instrument (model: DTG-60 H, company SHIMADZU Corporation (Japan) at the Department of Material Science and Engineering, Adama Science and Technology University, Adama.

## Result and discussion

### Sugarcane bagasse and ash characterizations

#### Proximate analysis

The proximate analysis of sugarcane bagasse was performed using wet basis analysis to identify the Physicochemical properties and components. These preliminary analytical results are typically one of the most important factors for determining the quality of solid substances. The calculation has been analyzed based on ASTM standards presented in Appendix A.

**Table 2:** Sugarcane bagasse proximate analysis result.

Proximate analysis	Result (%)
Moisture content	25.16
Volatile matter content	57.33
Ash content	8.94
Fixed carbon contents	8.57

Table 2 shows the proximate analysis of SCB. The sugarcane bagasse has an average moisture content of 25.16%, which is higher and not a permissible amount for ash generation. It has a significant impact on the quality of the ash during combustion. Higher moisture content in sugarcane bagasse lowers its caloric value, decreasing conversion efficiency and system performance because more energy is required to evaporate the moisture during burning. As a result, drying is required before combustion.

The findings presented in Table 2 indicate that bagasse contains a higher volatile matter content compared to other components, facilitating easier combustion and resulting in lower fixed carbon levels. This characteristic enhances silica formation in the resultant ash and reduces the heat required for reactions during combustion. Notably, the ash content of sugarcane bagasse was measured at 8.94%, which aligns closely with the 8.4% reported by^18^.

According to^18^, the moisture content of SCB is 24.0% and the ash content is 8.4%, thus this result is comparable with this work. The fixed carbon content has been comparable to the figure published by^19^, who found a fixed carbon value of 15.4%. The volatile matter content differs from the findings of these two researches (45.25% for the first and 67.8% for the second).

The observed deviations in composition can be attributed to various factors, including geographical location, climate, and weather conditions. Additionally, differences in biomass preparation, utilization methods, and storage techniques can significantly influence the characteristics of the materials. For effective silica synthesis, the ash must possess low carbon content while being rich in both ash and volatile matter. Under optimal conditions, such as, ash can yield high-purity silica, making it a valuable resource for various applications. Ensuring the right balance of these properties is crucial for maximizing the efficiency and quality of silica extraction from biomass wastes. Therefore, based on the aforementioned findings, the bagasse’s fixed carbon and ash concentration is favorable for silica synthesis.

#### Visual inspection

The SCB was burned under various settings, including changing the combustion temperature and duration. Table 3 indicates the color change of SCBA at different combustion temperatures and combustion times. The physical appearance of the ash was dark at low temperatures and time of combustion. It shows the poor quality of silica and the presence of a large number of carbons in the ash. As combustion temperature and time rose the ash color changed to white which indicates that the removal of carbon content in the ash and the black hue is caused by unburned carbon or melting of remaining metallic contaminants in the product surface.

**Table 3:** Visual characterization of bagasse ash at different temperatures and time.

No	Temperature (℃)	Time (hr.)	Observation
1	425	3	
2	500	2	
3	500	4	
4	650	1.5	
5	650	4.5	
6	800	2	
7	800	4	
8	875	3	

### Statistical optimization of silica yields

#### Modeling of silica synthesis

After the complete drying of the silica gel, the weight of dried silica for each run was measured and the yield was obtained by using Eq. (3) for each run of the experiment and presented in Table 4.

**Table 4:** Experimental values of silica yield.

Std	Combustion temperature (℃)	Combustion time (Hour)	Digestion time (Hour)	Yield (%)
1	500	2	1	27
2	800	2	1	77
3	500	4	1	42
4	800	4	1	83
5	500	2	3	39
6	800	2	3	82
7	500	4	3	52
8	800	4	3	85
9	425	3	2	23
10	875	3	2	83
11	650	1.5	2	70
12	650	4.5	2	82
13	650	3	0.5	61
14	650	3	3.5	73
15	650	3	2	75
16	650	3	2	76.5
17	650	3	2	77.5
18	650	3	2	78
19	650	3	2	76
20	650	3	2	76.5

**Table 5:** Sequential model sum of squares.

Source	Sum of Squares	Mean Square	F-value	*p*-value	
Mean vs. Total	89780.00	89780.00			
Linear vs. Mean	5700.10	1900.03	21.34	< 0.0001	
2FI vs. Linear	87.38	29.13	0.2832	0.8366	
**Quadratic vs. 2FI**	**1313.12**	**437.71**	**183.12**	**< 0.0001**	**“Suggested”**
Cubic vs. Quadratic	14.90	3.72	2.48	0.1533	Aliased

#### Fit Summary

“Sequential Model Sum of Squares” used to choose the highest order of polynomial in which the new terms are important and the model is not aliased. The significance of the model (Table 5) shows that the Quadratic model has the largest F-value and the p-value below 0.0001, so the Quadratic model is the most significant.

**Table 6:** Lack of fit test of model.

Source	Sum of Squares	Df	Mean Square	F-value	*p*-value	
Linear	1418.57	11	128.96	110.54	< 0.0001	
2FI	1331.19	8	166.40	142.63	< 0.0001	
**Quadratic**	**18.07**	**5**	**3.61**	**3.10**	**0.1201**	**Suggested**
Cubic	3.17	1	3.17	2.72	0.1603	Aliased
Pure Error	5.83	5	1.17			

#### B) lack of fit tests

“Lack of Fit Tests” (Table 6) used to show the chosen model that have an insignificant value of lack of fit. Since the lack of fit P-value is greater than 0.05 the lack of fit of quadratic model is insignificant. As a result, the model is suggested.

**Table 7:** Models’ summary statics.

Source	Std. Dev.	*R*²	Adjusted *R*²	Predicted *R*²	PRESS	
Linear	9.44	0.8001	0.7626	0.6950	2172.80	
2FI	10.14	0.8123	0.7257	0.4831	3682.51	
**Quadratic**	**1.55**	**0.9966**	**0.9936**	**0.9744**	**182.15**	**Suggested**
Cubic	1.22	0.9987	0.9960	0.8749	891.37	Aliased

#### Model summary statistics

“Model Summary Statistics” concentrates on the model with the highest “Adjusted R-squared” and “Predicted R-squared.” The projected R-squared reflects the model’s factor closeness, and the quadratic model was utilized to fit in this investigation. In terms of correlation coefficients, the quadratic formulation has the smallest standard deviation and the highest Adjusted and Predicted R-squared. As observed in Table 7, the deviation between adjusted and Predicted R-squared is less than 0.2 which indicates the appropriateness of the model. The cubic model is aliased, which implies that the effects of every parameter on the signal formation become indistinguishable.

**Table 8:** Fit summaries.

Source	Sequential *p*-value	Lack of Fit *P*-value	Adjusted *R*²	Predicted *R*²	
Linear	< 0.0001	< 0.0001	0.7626	0.6950	
2FI	0.8366	< 0.0001	0.7257	0.4831	
**Quadratic**	**< 0.0001**	**0.1201**	**0.9936**	**0.9744**	**Suggested**
Cubic	0.1533	0.1603	0.9960	0.8749	**Aliased**

#### Fit summary

In summary, the Quadratic model was selected to fit the regression of each variable factor and its response values.

Table 8 shows the results of an analysis of variance (ANOVA) to determine the significance Quadratic model. The P-values values were used as a means of checking the significance of each coefficient, as well as the intensity of each parameter’s interaction.

### ANOVA analysis for the quadratic model

#### Response 1: silica yield

As shown in Table 9, the model’s F-value of 330.08 shows that the fitting has enough signals and is significant. An F-value of this magnitude could only occur by chance with a 0.01% probability. Model terms with P-values below 0.0500 are considered significant, highlighting the importance of terms A, B, C, AB, AC, A², B², and C² in this context. Conversely, terms with P-values above 0.1000 are deemed insignificant. If the model includes many irrelevant terms (apart from those necessary for maintaining hierarchy), reducing the model could enhance its effectiveness. The F-value of 3.10 indicates that the lack of fit is not significant when compared to pure error, suggesting a 12.01% likelihood that noise could produce a significant Lack of Fit F-value. An insignificant lack of fit is advantageous in modeling.

**Table 9:** ANOVA analysis.

Source	Sum of Squares	Mean Square	F-value	*p*-value	
**Model**	7100.60	788.96	330.08	< 0.0001	Significant
A-Combustion Temp.	5202.00	5202.00	2176.37	< 0.0001	
B-Combustion time	269.12	269.12	112.59	< 0.0001	
C-Digestion time	228.98	228.98	95.80	< 0.0001	
AB	66.13	66.13	27.66	0.0004	
AC	21.13	21.13	8.84	0.0140	
BC	0.1250	0.1250	0.0523	0.8237	
A²	1069.51	1069.51	447.45	< 0.0001	
B²	1.73	1.73	0.7225	0.0052	
C²	221.08	221.08	92.49	< 0.0001	
Residual	23.90	2.39			
Lack of Fit	18.07	3.61	3.10	0.1201	not significant
Pure Error	5.83	1.17			
Cor Total	7124.50				

Since the gradients are less than 0.2, the Predicted R^2^ of 0.9744 logically agrees with the Adjusted R^2^ of 0.9936. Adequate precision measures the signal-to-noise ratio. A ratio of at least 4 is ideal. The ratio of 57.6174 indicates an adequate signal with enough flexibility to fit the regression equation. Furthermore, to verify the investigation’s correctness and repeatability, the coefficient of variation (C.V %) determines the overall experimental error as a percentage of the entire mean. Finally, I believe that this model is acceptable since it can be utilized for exploring the design space.

The regression coefficients related to 95% confidence intervals (CI) with Higher and Lower values are shown in Table 10. If zero falls between the High and Low 95% CI ranges, the factors have no or little impact. The coefficients for A, B, C, and the interaction terms AB and AC significantly influence silica production, as indicated by the 95% confidence intervals, which all share the same sign and do not include zero between their upper and lower bounds.

The coefficients of A, B, C, and the interaction terms AB and AC have significant effects on the production of silica since the 95% CI has the same sign and no zeros between the higher and lower CI. According to the result presented in Table 10, the low 95% CI for BC was (-1.34) and the high 95% CI was (1.09), this shows that the interaction effect of BC is insignificant or low.

**Table 10:** Regression coefficient and corresponding 95% coefficient interval.

parameters	Estimated Coefficient	Df	Error	Lowest 95% CI	Highest 95% CI	VIF
Intercept	76.52	1	0.6177	75.14	77.89	
A-Combustion Temperature	20.40	1	0.4373	19.43	21.37	1.0000
B-Combustion time	4.64	1	0.4373	3.67	5.61	1.0000
C-Digestion time	4.28	1	0.4373	3.31	5.25	1.0000
AB	-2.88	1	0.5466	-4.09	-1.66	1.0000
AC	-1.63	1	0.5466	-2.84	-0.4071	1.0000
BC	-0.1250	1	0.5466	-1.34	1.09	1.0000
A²	-10.19	1	0.4816	-11.26	-9.11	1.00
B²	-0.4094	1	0.4816	-1.48	0.6637	1.00
C²	-4.63	1	0.4816	-5.70	-3.56	1.00

Based on the factor parameters, the coefficients were adjusted by average effects. The similarity of the results from the experiments and the regression is expressed in terms of orthogonality. When the factors are orthogonal, the VIFs are 1. When the factors exhibit multicollinearity, the Variance Inflation Factors (VIFs) exceed 1. A higher VIF indicates a stronger correlation among the factors. Generally, VIFs below ten are deemed acceptable. All of the components are orthogonal since all of the VIFs are equal to one, as seen in the table above and they are acceptable.

### Model equation development

The model equation for silica synthesis from sugarcane bagasse that relates the response (yield) to the process parameters in the form of coded factors is presented in Eq. (6).

### “Final equation in terms of coded Factors”

6 $$\:Yield\:\left(Y\right)=77.04+30.68A+4.40B+3.76C-2.37AB-1.87AC-10.99{A}^{2}-0.5419{B}^{2}-4.54{C}^{2}$$

Where; A is combustion temperature; B is combustion time; C is digestion time.

Using the equation in terms of coded factors, one can make predictions about responses for certain levels of each factor. The high levels were coded as + 1 by default, while their low levels were coded as -1. The coded equation can be used to compare the factor coefficients and estimate the factors’ relative effects. By utilizing the equation based on coded factors, predictions can be made regarding responses at specific factor levels. The high levels are conventionally assigned a code of + 1, while the low levels are coded as -1. This coded equation allows for comparison of factor coefficients and estimation of their relative impacts.

### Predicted vs. actual values

**Fig. 4:**
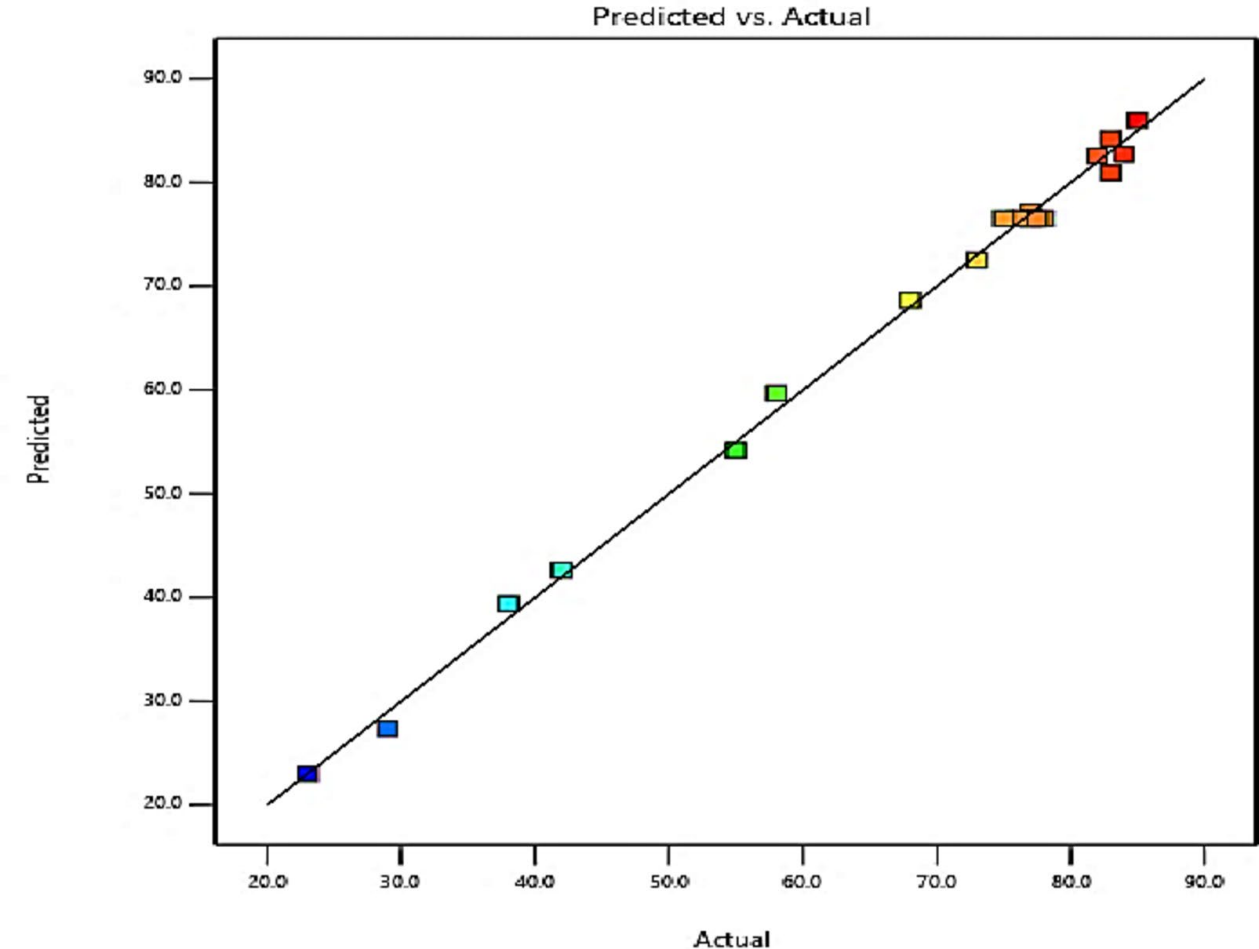
Actual vs. Predicted graph.

As shown in Fig. 4 the plot of predicted vs. actual value developed from the regression model equation indicates that the points follow a linear line which implies the model equation successfully expressed experimental results.

### Effect of process parameters on silica yield

Silica yield was affected by various process variables starting from sample preparation to drying the factors that are discussed in this study were Combustion Temperature, combustion time, and digestion time. They have a considerable impact on yield, as illustrated in the statistics below. These three variables are critical in the manufacturing of high-quality silica. Those three process variables influenced the yield of silica significantly. The effect of each parameter is discussed below.

### Combustion temperature

Increased combustion temperatures break down the unburned organic component which increases the silica content of the bagasse ash. As the ash silica content increased directly the yield of silica also increased.

**Fig. 5:**
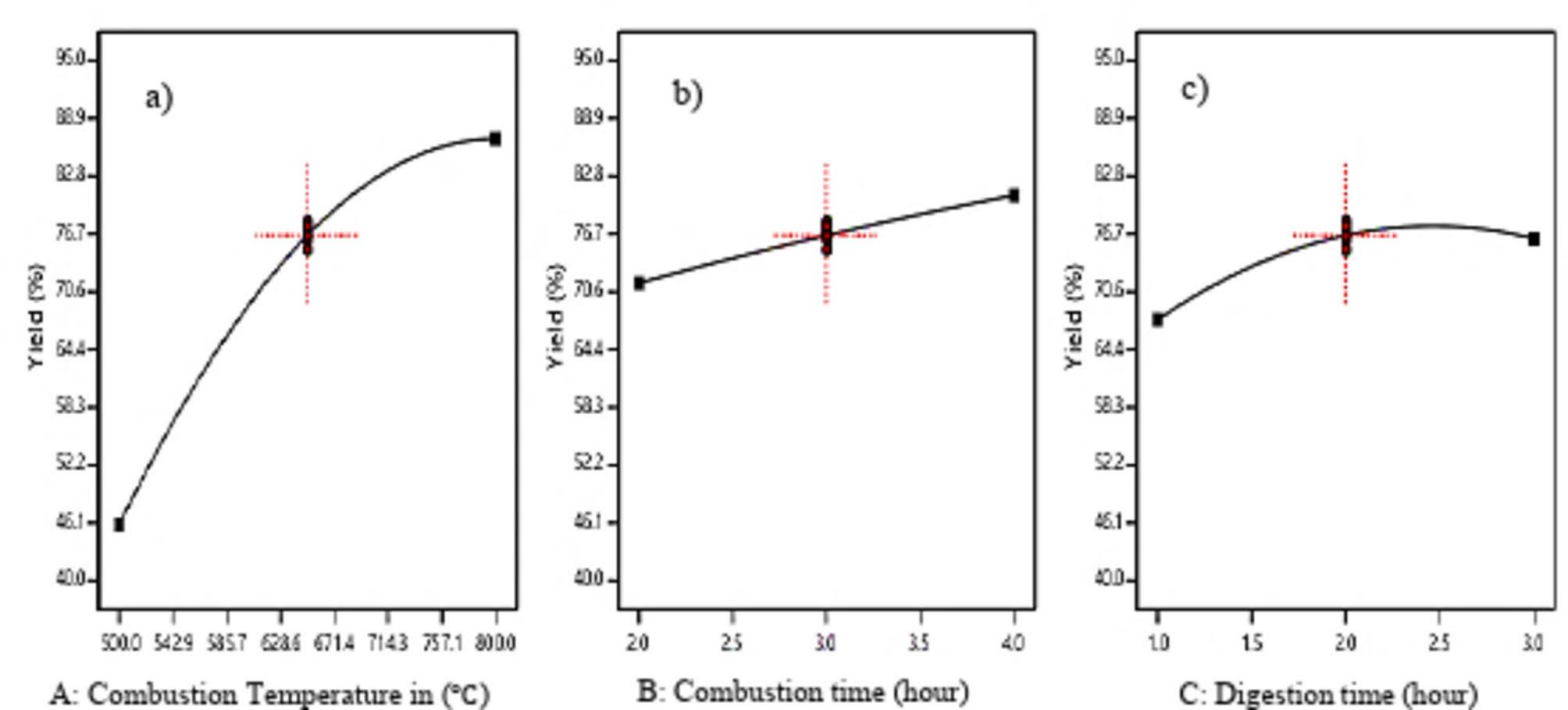
Single effects of (**a**) combustion temperature (**b**) combustion time (**c**) digestion time on silica yield.

As shown in Fig. 5(a), silica yield increased directly with the increase in Combustion temperature. The reason why this happened is that silica is mostly found in inorganic connections in sugarcane bagasse, but it is also covalently bound to organic molecules. This silica remains undissolved in hydroxide and is capable of withstanding high temperatures. Once the organic material from the SCB is eliminated, the inorganic residue can be relatively pure, leading to increased silica availability. However, extremely high combustion temperatures are not advised to produce amorphous silica. It can convert amorphous silica to crystalline silica.

### Combustion time

Figure 5(b) indicates the impact of incineration time on synthesis yield. Increasing the quantity of incineration duration greatly enhanced the percentage of silica synthesis. It is due to the removal of organic components or carbon content in the bagasse ash which maximizes the amount of silica present in the ash, which boosts the yield significantly.

### Digestion time

The influence of digestion time on silica yield is depicted in Fig. 5(c). Increasing the quantity of digesting time greatly improves the silica yield. Small reaction time leads to low conversion of raw material or low extraction of silicate from the ash. Increasing the digestion time increases silica yield until a limited amount of time (2.5 h). The digestion time has a considerable effect on silica yield.

### Interaction Effect

**Fig. 6:**
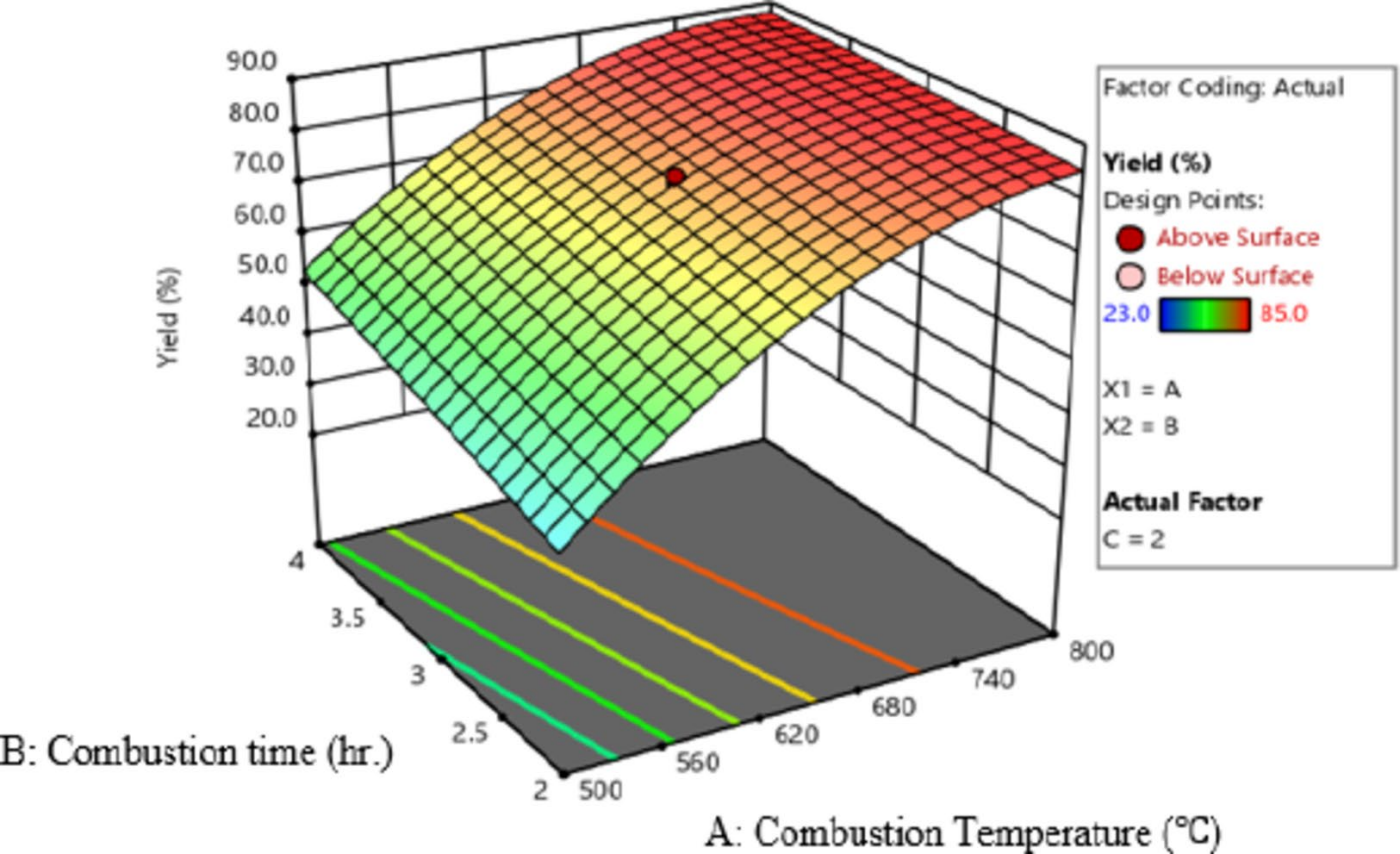
Surface plot of combustion temperature and combustion time.

The most frequent methods for displaying the interaction effects of process factors are the surface plot and the contour plot. Strong interaction has been discovered between the process variables. The 3D response surfaces illustrate the interactions between two variables while maintaining the third variable at its midpoint. Figure 6 displays the interaction effects of combustion temperature and combustion time.

Figure 6 depict the 3D surface plot of the interaction effect of combustion temperature and combustion time on silica yield. At higher combustion temperatures and times, a larger percentage of conversion was found. This could be due to the breakdown of organic matter which increases the proportion of silica in SCBA. In other words, it leads to enhanced silica yield.

**Fig. 7:**
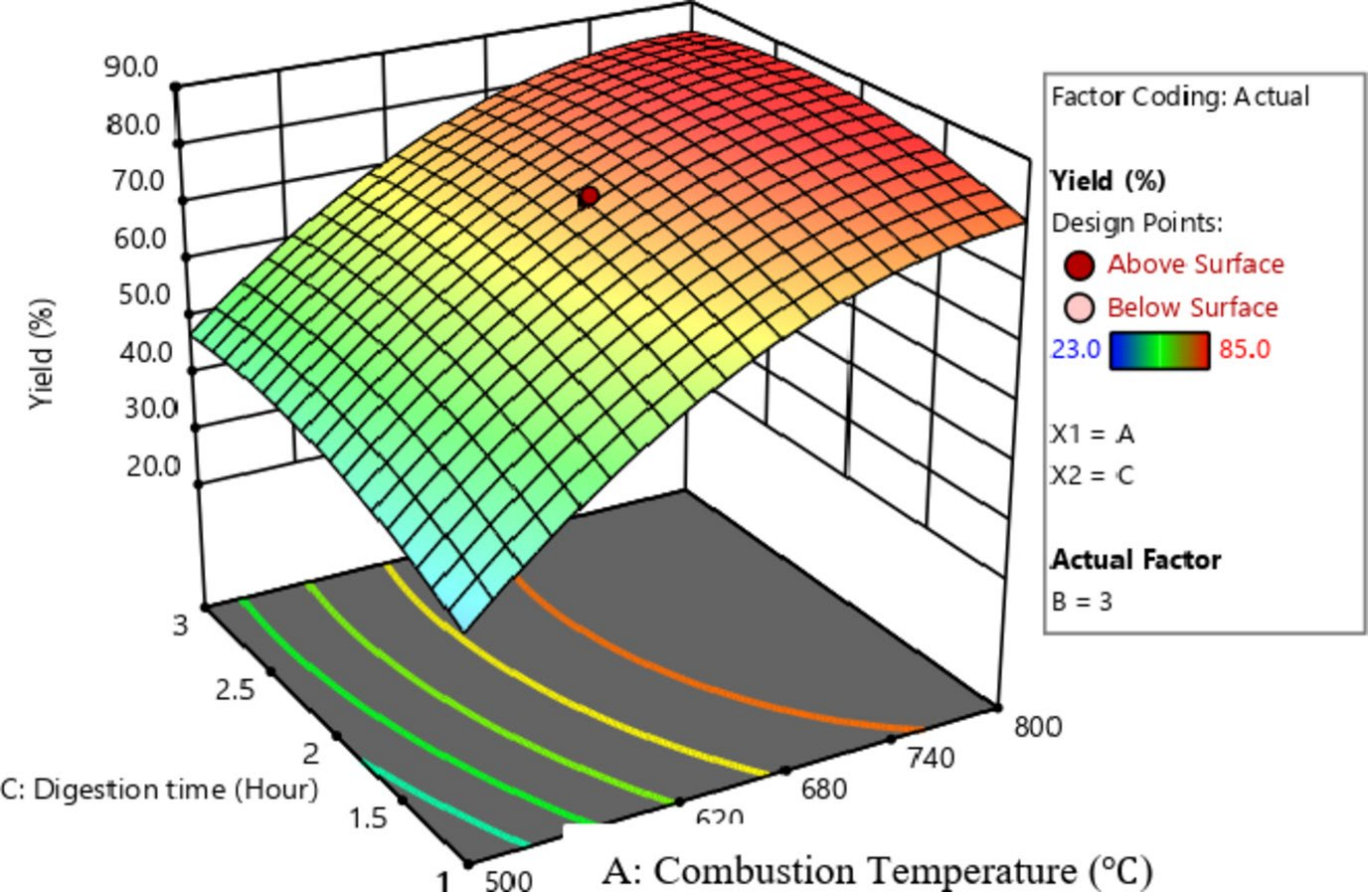
Surface plot of combustion temperature and digestion time.

Figure 7 presents a 3D surface plot illustrating the combined influence of combustion temperature and digestion time on silica yield. The data indicate that increasing both the combustion temperature and digestion duration enhances silica production. As observed in the Figures, maintaining digestion time at its midpoint while increasing combustion temperature and time results in a higher conversion percentage compared to scenarios with elevated digestion time and combustion temperature. This observation is further corroborated by the ANOVA results in Table 9, which highlight combustion temperature and time as the most critical variables affecting yield, as evidenced by their highest F-values.

The 3D surface plot illustrates that optimal yield occurs when both combustion temperature and duration are elevated, while digestion time is maintained at its midpoint. This suggests that combustion temperature and duration are the key process variables influencing yield, as indicated by their highest F-values in the ANOVA analysis.

### Optimization of silica synthesis from sugarcane bagasse

**Fig. 8:**
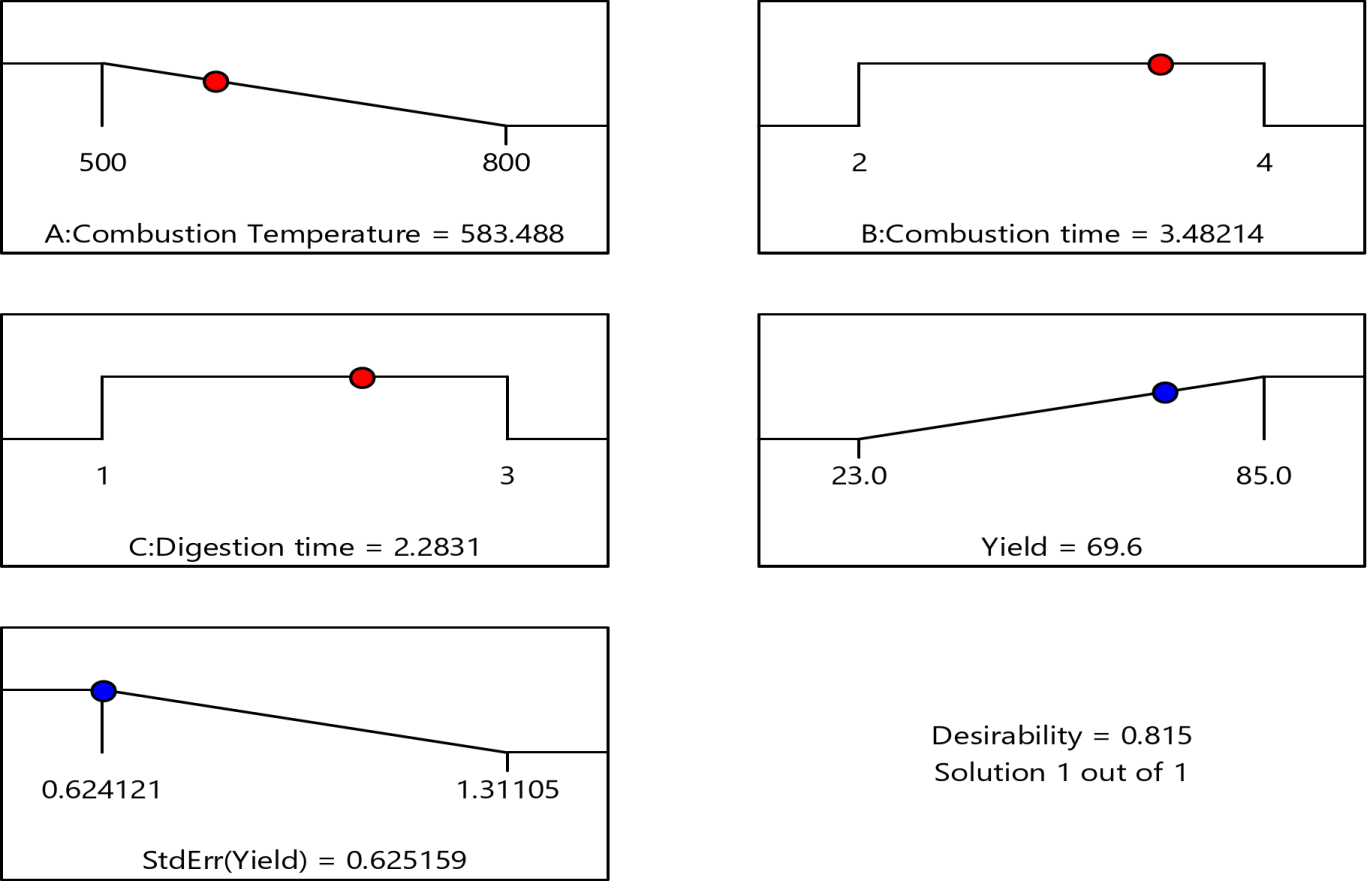
Ramps points of the optimization of silica yield.

The data obtained from statistical analysis show that the impact of the three process parameters and their combined effects on silica synthesis. This statistical optimization aims to get a high yield at low temperature, and combustion time with low extraction times. As a result, using the formulated regression model (quadratic model), process variables to get the maximum silica yield have been optimized by the design expert software. The optimum percentage of silica yield was obtained at the predicted combination of combustion temperature of 583.488 ℃, Combustion time of 3.482 h, and digestion time of 2.283 h. At these parameters, the model forecasted 69.6% and had a desirability level of 0.815. The optimization results have been presented on the ramp diagram in Fig. 8. To validate the model accuracy triplicated experiments were performed at the optimum condition and a 68.5% yield was obtained which has an error of 1.5%. The error (deviation) between the actual and the theoretical value of less than 5% is acceptable and the modeling is valid. As a result, this study demonstrates that sugarcane bagasse can be used for the synthesis of high-quality silica with a maximum yield at the optimum combustion temperature. The produced silica with 80.5% yield from agricultural waste of sugarcane bagasse ash^20^ and 45% of the maximum yield from sugarcane bagasse ash^21^. The result obtained in this work is highly comparable with other research work done on agricultural silica sources.

The synthesis of silica from sugarcane bagasse at the optimum point give advantage over the conventional silica production methods in its environmental impact and energy consumption. The synthesis amorphous silica by smelting powder quartz at a temperature of 1450 ℃ which was higher than the temperature used to combust SCB in this research^22^. As^13^, stated, the Silica extraction from sand or quartz demands high energy compared with agricultural sources. The produce amorphous silica by burning sugarcane bagasse black ash at 600 ℃ for 3 h get 45% of silica yields^23^. According to^3^, produce silica by combustion of weed Saccharum ravannae leaves at temperature of 700 ℃ for 3 h and obtains yield of 35%. The statical optimization in this research give scientific analysis which give statical model and optimal synthesis condition that produce high yield of silica at low temperature. Since, the operation temperature have a direct energy consumption of the furnace, we conclude that the silica synthesis from sugarcane bagasse is more energy efficient than that of conventional sources.

### Synthesized silica characterization

To validate the quality and properties of the silica produced under optimum conditions, XRD, FTIR, SEM, and TGA analytical techniques were used. These characterization methods collectively provide a comprehensive understanding of the silica produced under the specified optimal conditions, confirming its structural, chemical, morphological, and thermal properties.

#### XRD characterization

**Fig. 9:**
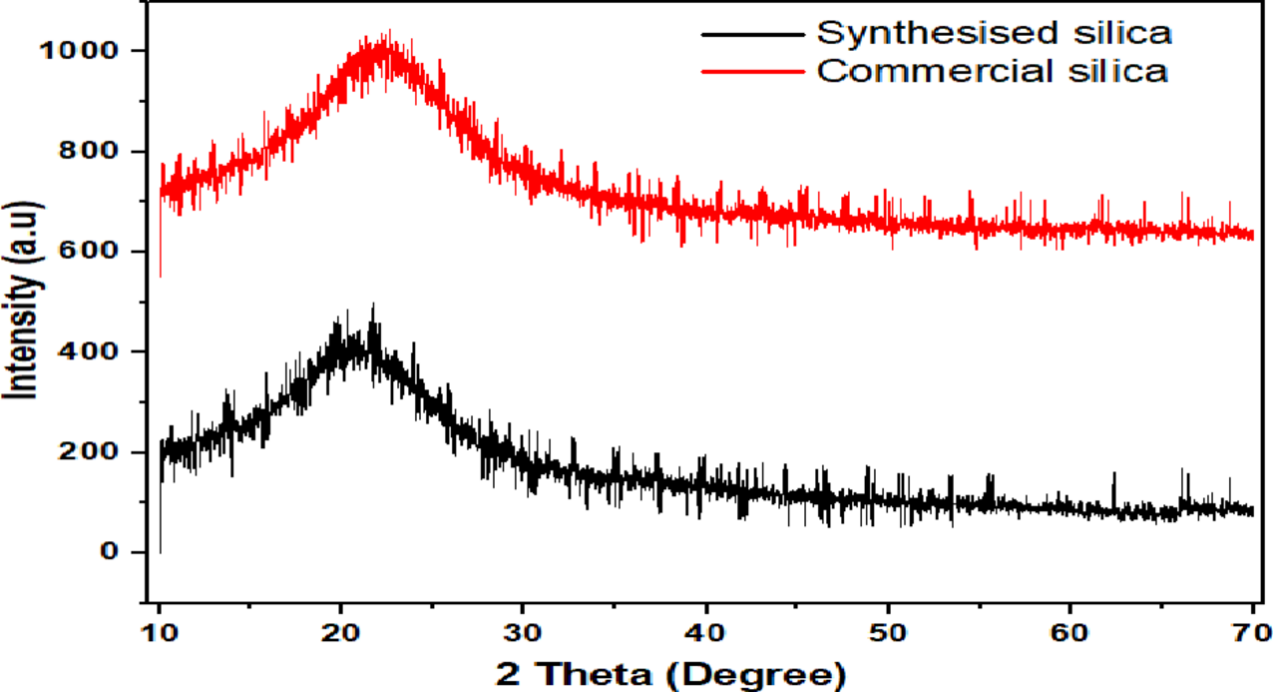
XRD analysis of commercial and synthesis silica at various conditions.

The XRD analysis of synthesized silica presented in Fig. 9, demonstrates the absence of crystalline minerals. To validate the quality and crystallites of the produced silica, XRD analysis of commercial silica (98% purity) was used for validation. As noted by^24^, amorphous materials are characterized by a prominent hump in the diffraction pattern between 15 and 35°. In Fig. 9, a broad hump is observed between 18 and 27° of 2θ, aligning with previous findings on amorphous silica. This suggests that the silica possesses a completely amorphous structure due to its disordered network. The silica has a significant peak occurring at 2θ of 22.38°. The XRD pattern in Fig. 9 confirms that the synthesized silica exhibits an amorphous morphology, consistent with results from^25^. Notably, the XRD analysis of synthesized silica reveals a similar hump shape at the same angles as that of commercial silica. This reveals the synthesis of silica from sugarcane bagasse has high quality and similar morphology. It agrees with the result presented by^9^, for the synthesis of silica from agricultural wastes.

The crystallite index obtained from the origin XRD graph analysis, the crystalline area was 1831.7 units square and total areas of 4987.6 units’ square. Therefore, by using Eq. (5) the crystalline index becomes 26.213%. A crystalline index below 35% indicates that the substance has an amorphous nature.

### FTIR spectroscopy

**Fig. 10:**
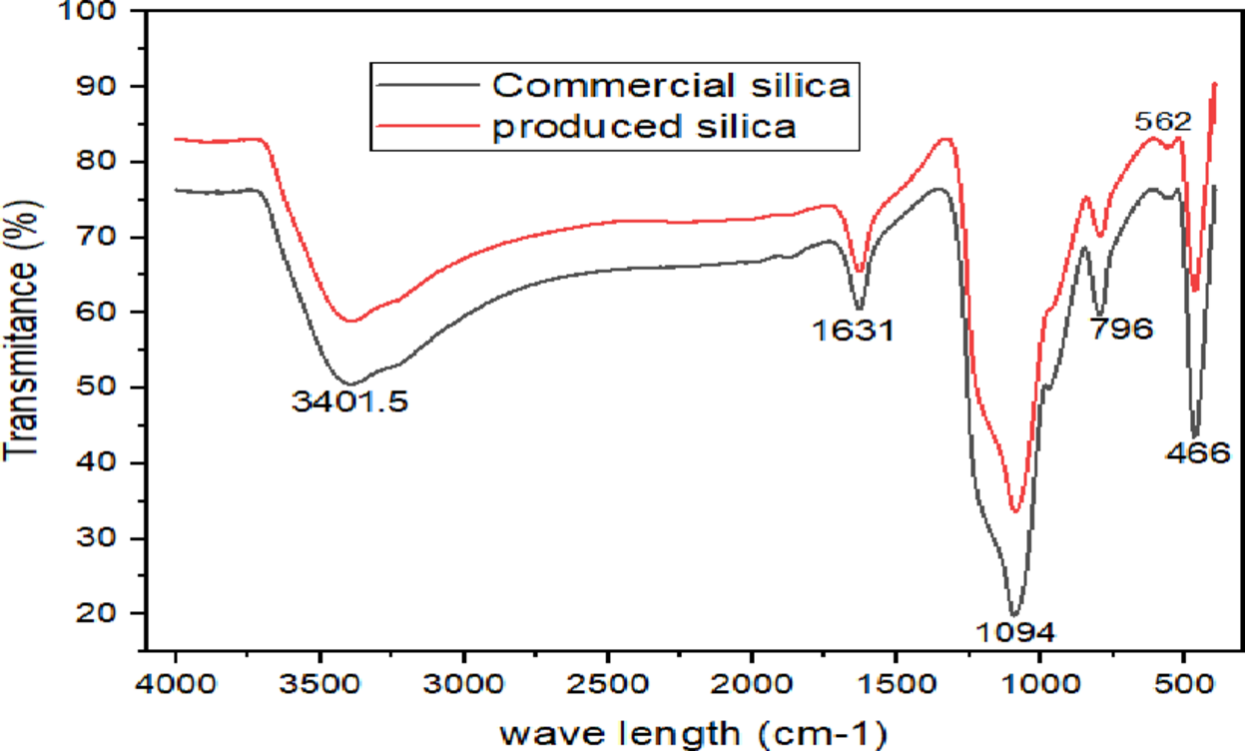
FTIR spectra for commercial and produced silica.

Figure 10 shows the FTIR spectroscopy analysis of synthesis silica and commercial silica. The characteristic and peak position of the FTIR spectra for developed silica agrees well with the FTIR spectra of commercial silica. As^26^, depict, the bands at 466 cm^− 1^ are responsible for Si-O-Si bending vibration, the Wavenumber (WN) of 796.5 cm^− 1^ responsible for symmetric stretching vibrations of Si–O–Si and WN of 467 cm^− 1^ represent out-of-plane Si-O-Si deformations vibration. According to^27^, asymmetric stretching vibrations of Si-O-Si are responsible for the largest spectral area and associated shoulders (1300–1000 cm^[–1^).

The most significant identified spectral region concerning silicon as lying between 1020 and 1100 cm⁻¹ are linked to the asymmetric stretching vibrations of Si-O-Si bonds^21^. Consequently, the wavenumber of 1094 cm⁻¹ illustrated in Fig. 10 represents Si-O-Si bonds asymmetric stretching. In a related study^26^, note that the wavenumber at 1631 cm⁻¹ corresponds to the bending or deformation mode associated with water molecules integrated within the Si-O-Si framework. Additionally, the spectral bands observed between 3500 and 3300 cm⁻¹ are indicator of oxygen-containing silanol groups (Si-OH). Thus, the peak identified at 3401 cm⁻¹ is associated with hydroxyl (OH) groups. The broader bands in the range of 3000 to 3500 cm⁻¹ across all materials are attributed to the stretching vibrations of silanol groups on the silica surface and the O-H groups from water molecules trapped within the porous structure^28^.

As illustrated in Fig. 10, the FTIR spectra of both commercial silica and silica derived from bagasse exhibit comparable peak patterns. This observation corroborates the XRD results, which indicate that the synthesis silica has high purity and amorphous morphology, meeting the standards for commercial-grade materials.

**Fig. 11:**
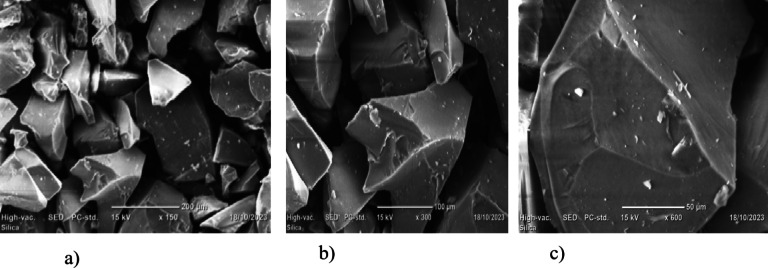
SEM analyses of silica at different magnifications (**a**) 150X (**b**) 300X (**c**) 600X.

#### Scanning electron microscope image analysis

The scanning electron microscope (SEM) is a powerful tool used to observe the surface structures of specimens at much higher magnifications than light microscopes can achieve. SEM operates by scanning a focused beam of electrons over a sample, and the electrons interact with the atoms in the sample, producing various signals that can be detected and converted into images. Figure 11 shows SEM images at various resolutions of silica synthesized from sugarcane bagasse. It revealed that the particles have irregular surfaces and shapes. This morphology is comparative to the silica gel morphology reported by^12,24^.

As shown in Fig. 11 (a) at 150X magnification, the SEM provides a relatively low magnification of the specimen. At this magnification level, the overall surface texture and structure of larger features, such as the shape of a material or large particles, large cracks, pores, or grains that may be present on the surface. In Fig. 11 (b) the SEM at 300X magnification shows greater details of the surface characteristics, including smaller surface abnormalities such tiny fractures, pores, or material grain boundaries. The surface composition of materials and surface texture features become more noticeable. Similarly, Fig. 11 (c) at 600X magnification SEM begins to display the specimen’s fine details bringing to light even more minute characteristics that are not discernible at lower magnifications, including the microscopic surface details. The ability to view surface fissures, tiny topography features, individual nanostructures, and particulate or submicron-sized features provides a far higher resolution view of the texture and structure of materials.

### Brunaure Emmet Teller (BET) analysis

**Fig. 12:**
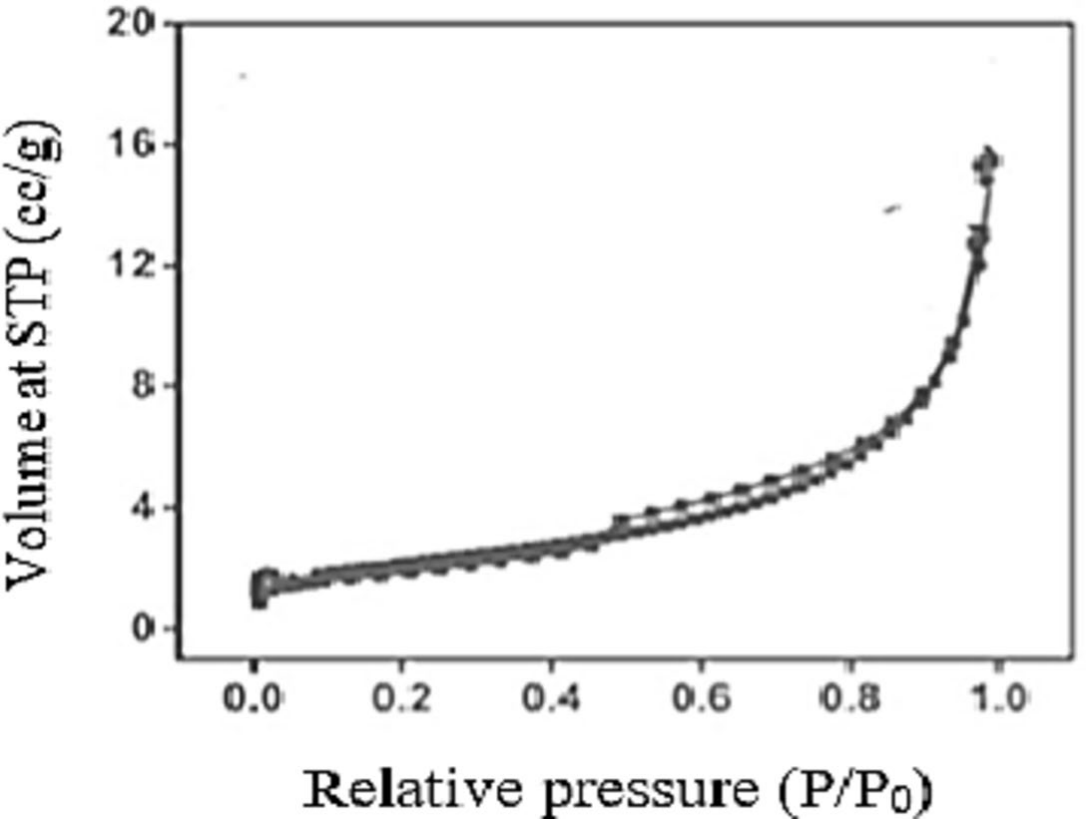
BET surface area plot from nitrogen adsorption isotherms form produced silica.

The BET analysis was conducted on mesoporous silica to determine the tested sample surface area and pore properties. The mass content of the sample had an impact on the surface area, pore radius, and pore volume as determined by BET in Fig. 12. Therefore, the pore properties of the mesoporous silica made with sugar cane bagasse as the raw material are shown in Table 11. According to the BET analysis, the synthesized SCBA-based mesoporous silica had specific surface area of 512.474 m²/g, pore volume of 0.524 cc/g, and pore radius of 23.57 Å, respectively. The synthesized silica has a BET surface area of 200–800 m²/g, a BJH pore volume of 0.5–0.89 cm³/g, and a pore size of 2–50 nm, which makes it ideal for various applications^29^. These findings confirmed that the resulting porous material falls within the category of mesoporous materials.

**Table 11:** Physical properties of SCBA Mesoporous samples obtained from N2 adsorption measurements.

Physical Properties	Value
Surface Area (m^2^/g)	512.474 m²/g
Pore Radius (Å)	23.57 Å
Pore Volume (cc/g)	0.524 cc/g

### Thermo-gravimetric analysis (TGA)

**Fig. 13:**
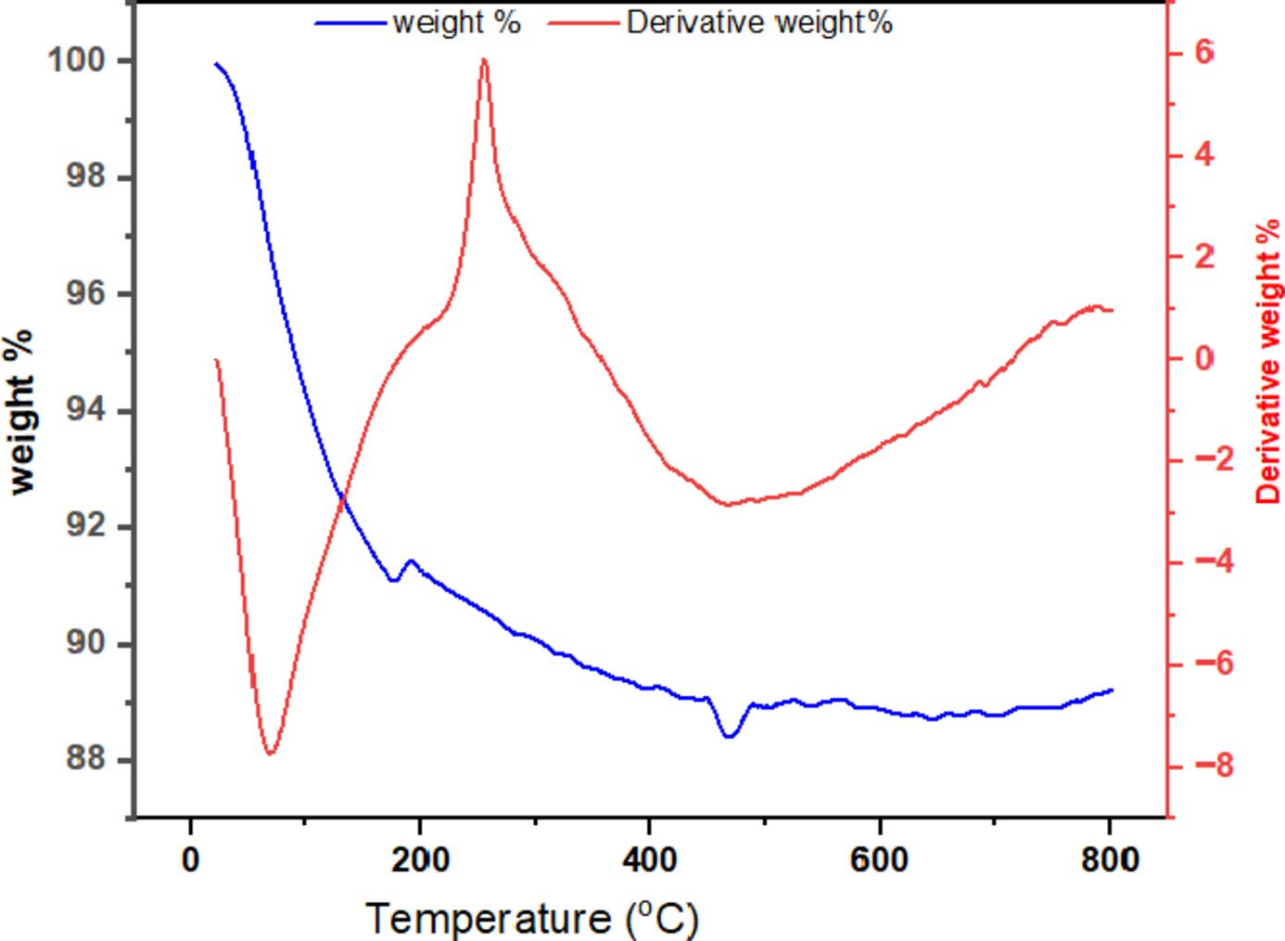
TGA analysis of silica.

The Thermo-gravimetric Analysis (TGA) of the produced silica, as depicted in Fig. 13, provides critical insights into its thermal stability, which is an essential characteristic for various applications. Based on the DTA graphs (differential mass loss graph) in Fig. 13 the weight losses have three main stages. The rapid weight loss from room temperature to 200 ℃ represents the dehydration of physically adsorbed water molecules at the silica surface^30^. In this stage, 8.81% weight was lost. The second stage from a temperature range of 200 to 400 °C shows, weight loss due to the removal of high volatile matter and chemically adsorbed water to the Si-OH group through a covalent bond^9^. In this stage, 1.72% mass loss was observed. The 3rd stage is the stable stage where very low mass loss occurs. It has happened in the temperature range of 450–800 ℃. As the TGA graph revealed the silica is highly thermal stable and good for high temperature area applications. Since the organic molecules were removed while calculations of silica there is very small mass loss during this stage and it shows high thermal stability and resistance at high temperature. However, unexpected ups and downs (weight gain and loss) were observed on the TGA curves. This happened due to the oxidation (mass gain) and reduction (mass loss) of the materials at specific temperatures.

## Conclusion

This research has effectively highlighted the viability of sugarcane bagasse as a promising source for producing high-purity silica. The proximate analysis, along with XRD and FTIR assessments of the ash, confirms that sugarcane bagasse can serve as an alternative silica source. Additionally, the use of organic acids such as citric acid notably improves silica yield while reducing contaminants.

The statistical analysis found a significant correlation between combustion, temperature, combustion time, digestion time, and silica yield which was represented by the quadratic model. The studies found that 69.6% optimum yield at a combustion temperature of 583.488 ℃, combustion time of 3.482 h, and digestion time of 2.283 h. It also shows that the combustion temperature and combustion time are predominant parameters that affect the silica yield. The XRD, FTIR, SEM, BET and TGA characterizations confirmed the quality and amorphous nature of the synthesized silica.

Optimizing the extraction of silica from sugarcane bagasse through statistical methods presents an effective strategy for improving both yield and efficiency. Utilizing techniques like response surface methodology allows us to pinpoint the best conditions for silica recovery, which plays a vital role in sustainable waste management and the creation of valuable materials. This approach not only enhances resource use but also fosters a circular economy by converting agricultural waste into high-value products, leading to new opportunities across different industries.

## Data Availability

The datasets generated and/or analyzed during the current study are not publicly available but are available from the corresponding author upon reasonable request.
